# Serum neurofilament light chain (sNfL) values in a large cross-sectional population of children with asymptomatic to moderate COVID-19

**DOI:** 10.1007/s00415-021-10554-1

**Published:** 2021-04-23

**Authors:** Tobias Geis, Susanne Brandstetter, Antoaneta A. Toncheva, Otto Laub, Georg Leipold, Ralf Wagner, Michael Kabesch, Severin Kasser, Jens Kuhle, Sven Wellmann, CoKiBa Study group, Bettina Aichholzer, Georg Mair, Michaela Wruk, Imke Reischl, Andreas Ambrosch, David Antos, Stephan von Koskull, Christian Becker, Elisabeth Beer, Hubert Schirmer, Georg Birkinger, Andreas Blueml, Heike Buntrock-Döpke, Mona Castrop, Jost Dieckerhoff, Renate Eichhorn, Dominik Ewald, Gudrun Fleck Alfred Heihoff, Jürgen Geuder, Jens Grombach, Peter Gutdeutsch, Florian Segerer, Thomas Habash, Sonja Habash, Susanne Harner, Christoph Herbst, Daniela Heuschmann, Meike Hofmann, Michael Horn, Birgit Jork-Kaeferlein, Monika Schwarz, Reinhard Hopfner, Guido Judex, Bastian Baumgartner, Monika Corbacioglu, Sabrina Lindner, Bettina Meinel, Alena Bauer, Hannes Löw, Annamaria Szulagyi-Kovacs, Sarah Laub, Annegret Klein, Cosima Koering, Niclas Landvogt, Claudia Soehngen, Karin Rasp, Gudrun Schick-Niedermeier, Marinus Laub, Otto Laub, Georg Leipold, Petra Schmid-Seibold, Johannes Pawlak, Michaela Reitz, Georg Puchner, David Peterhoff, Christiane Razeghi, Stefan Razeghi, Christine Rehe, Klaus Rehe, Matthias Scheffel, Ludwig Kaesbauer, Roland Schmid, Michael Strobelt, Nina Schoetzau, Andrea Schweiger-Kabesch, Marko Senjor, Michael Sperlich, Guenter Theuerer, Guenter Steidle, German Tretter, Victor von Arnim, Marlene Volz-Fleckenstein, Sebastian Einhauser, Patrick Neckermann, Natascha Borchers, Elisangela Santos-Valente, Parastoo Kheiroddin, Patricia Schöberl, Jakob Niggel, Stephan Gerling

**Affiliations:** 1 University Children’s Hospital Regensburg (KUNO), Hospital St. Hedwig of the Order of St. John, University of Regensburg, Steinmetzstr. 1-3, 93049 Regensburg, Germany; 2 Research and Development Campus Regensburg (WECARE), at the Hospital St. Hedwig of the Order of St. John, University of Regensburg, Regensburg, Germany; 3 Pediatric Office Dr. Laub, Rosenheim, Germany; 4 Pediatric Office Dr. Leipold, Regensburg, Germany; 5 Institute of Medical Microbiology and Hygiene, Molecular Microbiology (Virology), University of Regensburg, Regensburg, Germany; 6 Department of Pediatric Hematology and Oncology, University Children´s Hospital Basel (UKBB), University of Basel, Basel, Switzerland; 7 Neurology Clinic and Policlinic, MS Center and Research Center for Clinical Neuroimmunology and Neuroscience Basel, University Hospital Basel, University of Basel, Basel, Switzerland

**Keywords:** COVID-19, Children, Brain, Neurology, Neurofilament

## Abstract

**Background:**

Serum neurofilament light chain (sNfL) is an established biomarker of neuro-axonal damage in multiple neurological disorders. Raised sNfL levels have been reported in adults infected with pandemic coronavirus disease 2019 (COVID-19). Levels in children infected with COVID-19 have not as yet been reported.

**Objective:**

To evaluate whether sNfL is elevated in children contracting COVID-19.

**Methods:**

Between May 22 and July 22, 2020, a network of outpatient pediatricians in Bavaria, Germany, the Coronavirus antibody screening in children from Bavaria study network (CoKiBa), recruited healthy children into a cross-sectional study from two sources: an ongoing prevention program for 1–14 years, and referrals of 1–17 years consulting a pediatrician for possible infection with severe acute respiratory syndrome coronavirus 2 (SARS-CoV-2). We determined sNfL levels by single molecule array immunoassay and SARS-CoV-2 antibody status by two independent quantitative methods.

**Results:**

Of the 2652 included children, 148 (5.6%) were SARS-CoV-2 antibody positive with asymptomatic to moderate COVID-19 infection. Neurological symptoms—headache, dizziness, muscle aches, or loss of smell and taste—were present in 47/148 cases (31.8%). Mean sNfL levels were 5.5 pg/ml (SD 2.9) in the total cohort, 5.1 (SD 2.1) pg/ml in the children with SARS-CoV-2 antibodies, and 5.5 (SD 3.0) pg/ml in those without. Multivariate regression analysis revealed age—but neither antibody status, antibody levels, nor clinical severity—as an independent predictor of sNfL. Follow-up of children with pediatric multisystem inflammatory syndrome (*n* = 14) showed no association with sNfL.

**Conclusions:**

In this population study, children with asymptomatic to moderate COVID-19 showed no neurochemical evidence of neuronal damage.

## Introduction

Multiple differences have emerged between adults and children in the clinical manifestations of severe acute respiratory syndrome coronavirus 2 (SARS-CoV-2) infection, with notable respect to severity, outcome, and neurological involvement [1–3]. In addition, in children with coronavirus disease 2019 (COVID-19), a multisystem hyperinflammatory syndrome has recently been identified, termed pediatric multiorgan immune syndrome (PMIS), which shares many clinical features with Kawasaki disease [4], including a presumed immune-mediated post-infectious etiology [5].

Neurofilament light chain (NfL), a cytoplasmic protein exclusively expressed in central and peripheral nervous system neurons, has recently become established as a specific biomarker of neuroaxonal damage [6]. Raised levels of serum NfL (sNfL) have been found in numerous acute and chronic neurologic diseases, including in adults with mild-to-moderate and severe COVID-19 [7, 8]; levels in pediatric COVID-19 and PMIS have not as yet been reported.

Our aim in this study was to measure SARS-CoV-2 antibody and sNfL levels in a large pediatric population to determine the impact of COVID-19 on neuronal integrity.

## Materials and methods

### Study design and population

The Coronavirus antibodies in Kids from Bavaria (CoKiBa) study group collected samples for this cross-sectional study between May 22 and July 22 2020 in three regions of Southern Germany, which were strongly affected by the first wave of the pandemic. Using a sliding window approach CoKiBa invited participation by the parents of all the 1–14 years, they were scheduled to see in 2020 as part of a prevention program. They also invited any child, including siblings older than 14 years, who with their parents’ consent wished to take part. Exclusion criteria for participating in this sNfL study were preexisting chronic or congenital diseases. All data were collected using an online self-administered parental questionnaire. Entries were fully anonymized and only accessible to participants using an individual code on the Qnome platform (www.qnome.eu), as previously detailed [9]. The study was approved by the University of Regensburg institutional review board (20-1865-101), and written informed consent was obtained from the parents.

### SARS-CoV-2 antibody tests

The specific antibody response to SARS-CoV-2 was evaluated using two test kits: the commercially available licensed qualitative Elecsys^®^ Anti-SARS-CoV-2 assay (Roche Diagnostics, Rotkreuz, Switzerland; https://diagnostics.roche.com) and a validated in-house ELISA [10]. The Elecsys^®^ Anti-SARS-CoV-2 assay does not discriminate between the antibody type(s) present and can detect IgA, IgM, and IgG. The test is based on a recombinant nucleocapsid (N) antigen and has a cutoff value of 1.0 (S/Co). The in-house ELISA based on the SARS-CoV-2 S-protein receptor-binding domain quantifies total IgG and has a cutoff value of 1.0 (S/Co). All S/Co results < 1.0 were considered negative*.* S/Co antibody responses ≥ 100 in the Elecsys^®^ Anti-SARS-CoV-2 assay were defined as strong and participants were invited for clinical follow-up for PMIS.

### sNfL analysis

sNfL was measured at the same timepoint as Anti-SARS-CoV-2 using the digital NF-light™ single molecule array assay on the HD-X Analyzer (Quanterix, Lexington, MA), as described elsewhere [6].

### Statistical analyses

Descriptive statistics were calculated for the entire sample and both subgroups (children with and without SARS-CoV-2 antibodies). As sNfL values ranged widely, with considerable numbers of outliers, logarithmic transformation was used to normalize distributions. We used multivariable linear regression (MLR) models adjusted for age and sex to analyze associations between SARS-Cov-2 antibody status, antibody levels, PMIS symptoms and neurologic symptoms on the one hand and sNfL levels on the other. We performed all analyses using IBM SPSS Statistics 24.

## Results

A total of 2934 children were recruited, of whom 2832 (96.5%) were successfully tested for SARS-CoV-2 antibodies and had complete personal data. Samples sufficient for sNfL analysis were obtained from 2687 participants, 35 individuals were excluded due to preexisting chronic or congenital disease, including epilepsy (*n* = 12), cerebral palsy (*n* = 3), cystic fibrosis or chromosomal/genetic diseases (*n* = 10), inborn metabolic or endocrinological disorders (*n* = 5), oncologic, or inflammatory diseases (*n* = 5). Of the remaining 2652 participants, 148 (5.6%) were SARS-CoV-2 antibody-positive by virtue of an above-cutoff result in at least one of the two antibody tests. Table 1 lists the neurologic symptoms in SARS-CoV-2-positive participants. There were no significant age or sex differences between the antibody-positive and negative subgroups (Table 2). In 64 (43%) children the timepoint of COVID-19 onset was available as at last one SARS-CoV-2 PCR-positive nasopharyngeal swab was recorded from the children themselves (n = 25) or their parents (*n* = 39), which was on average 85 days (min 5, max 118, IQR 59–93 days) prior to blood sampling for SARS-CoV-2 antibodies and sNfL analysis.

**Table 1:** Symptoms and severity in SARS-Cov-2 antibody-positive children

Symptoms and severity, *n* (%)	Antibody-positive *n* = 148
None at all	38 (25.7)
No neurologic symptom	101 (68.2)
Headache	33 (22.3)
Dizziness	6 (4.0)
Muscle aches	24 (16.2)
Loss of smell	6 (4.0)
Loss of taste	7 (4.7)
Hospitalization for COVID-19	2 (1.3)

**Table 2:** Population characteristics stratified by SARS-CoV-2 antibody status

	All *n* = 2652	Antibody-positive *n* = 148	Antibody-negative *n* = 2504
Sex (female), *n* (%)	1280 (48.3)	73 (49.3)	1207 (48.2)
Age (years), M (SD)	7.1 (3.8)	7.5 (4.0)	7.1 (3.8)
sNfL, M (SD)	5.5 (2.9)	5.1 (2.1)	5.5 (3.0)

*sNfL* Serum neurofilament light chain

**Fig. 1:**
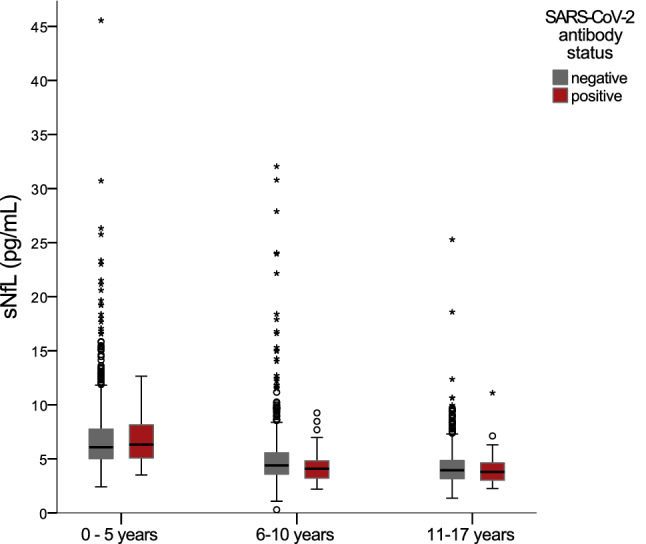
Association of SARS-CoV-2 antibody status (negative/positive) with sNfL. SARS-CoV-2 antibody status is not significantly associated with sNfL after adjusting for age and sex (linear regression analysis: B =  − 0.052, SE (B) = 0.030; *P* = .088. *n* = 2651)

The mean sNfL level in the total cohort was 5.5 pg/ml (SD 2.9); mean levels in the antibody-positive and negative subgroups were 5.1 (SD 2.1) pg/ml and 5.5 (SD 3.0) pg/ml. Multivariable MLR analysis revealed no significant association between SARS-CoV-2 antibody status and sNfL (Fig. 1).

**Fig. 2:**
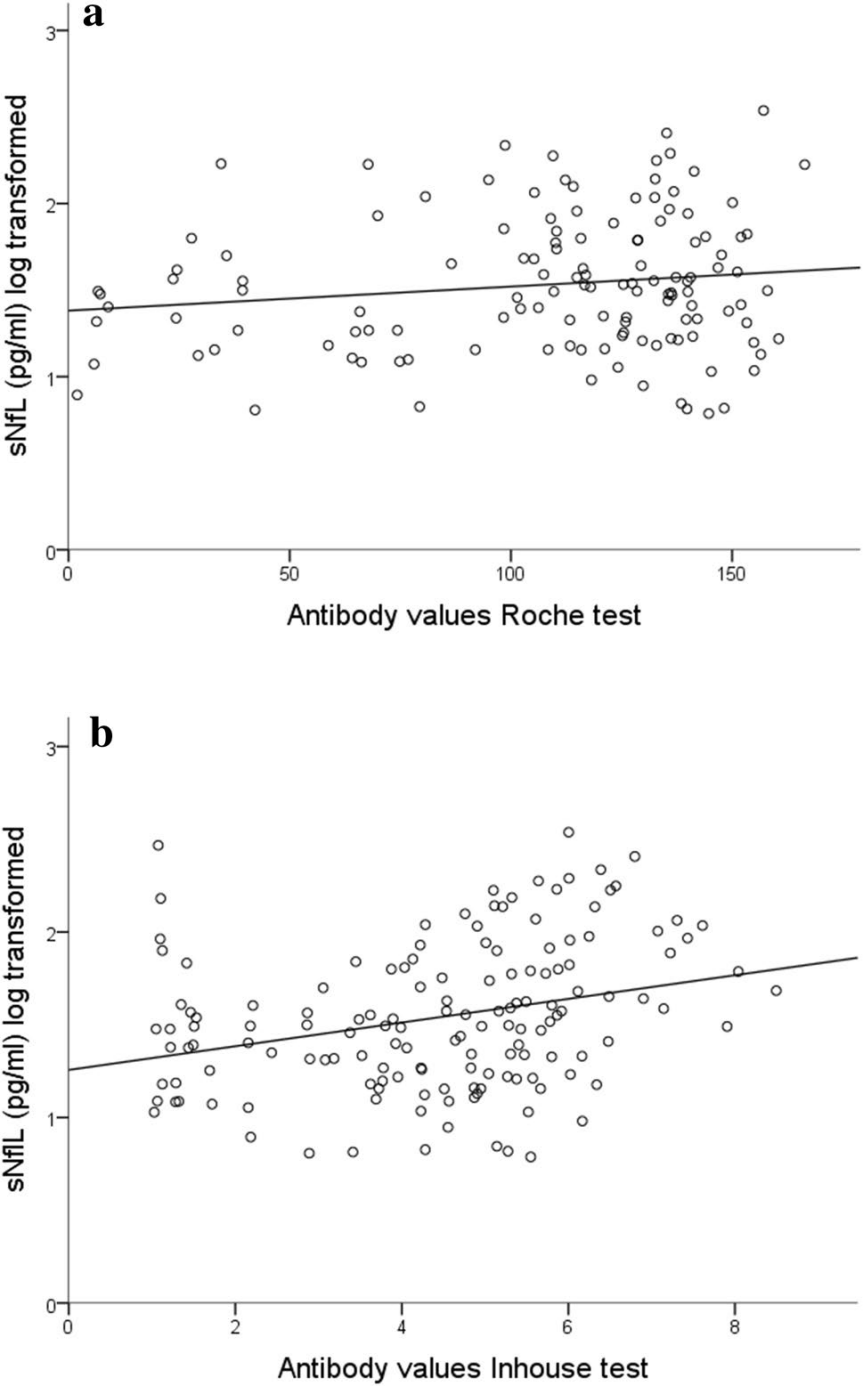
Association of SARS-CoV-2 antibody levels with sNfL. Antibody levels in the Roche test (**a**; *n* = 130) and inhouse test (**b**; *n* = 147) are not significantly associated with sNfL after adjusting for age and sex [linear regression analysis: **a** B = 0.001, SE (B) = 0.001; *P* = .310. **b** B = 0.022, SE (B) = 0.015; *P* = .144]. Antibody levels: all participants with a positive test result > 1

In the antibody-positive subgroup, we tested for correlation between antibody and sNfL levels separately for the Elecsys^®^ results (*n* = 130; Fig. 2a) and ELISA results (*n* = 147; Fig. 2b). MLR revealed no significant association in either analysis.

Children with SARS-CoV-2 antibody levels > 100 were followed up for possible PMIS: 14 of the 50 children were diagnosed with one or more PMIS-compatible symptoms. However, we found no association between PMIS symptoms and sNfL levels (regression coefficient B = 0.22; standardized regression coefficient beta = 0.099, *P* = 0.144).

Nor did we find any association, after adjusting for age and sex, between sNfL levels and the time elapsing from COVID-19 onset to blood sampling for SARS-CoV-2 antibodies (regression coefficient B < 0.01; standardized regression coefficient beta = 0.007, *P* = 0.948).

## Discussion

Our population study found no increase in the levels of sNfL, a highly specific biomarker for neuronal damage, in children with asymptomatic to moderate SARS-CoV-2 infection. There was no association with sNfL even in children with extremely high antibody levels, neurologic symptoms, or symptoms consistent with PMIS.

Early in the pandemic sustained sNfL elevation reflecting neurologic involvement was described in adults with severe COVID-19 admitted to intensive care [8, 11]. More surprisingly, raised levels were also found in adults with mild-to-moderate COVID-19 who were either neurologically asymptomatic or who exhibited only minor neurologic symptoms [7]. Our result is in line with findings in young adults < 35 years of age and suggests that children are less susceptible to neurologic involvement than older adults [1, 7]. Further evidence of age dependency is provided by the higher rate of neurologic complications in older COVID-19 patients [11].

PMIS presents with a wide spectrum of signs and symptoms [4] and is thought to occur as an exaggerated autoimmune response to infection between one to more than 9 weeks after COVID-19 [12]. A characteristic pattern of PMIS-associated cytokine storm and immune response has been identified [12], prompting the hypothesis that a specific hyperinflammatory response to SARS-CoV-2 infection causes the neuronal damage in PMIS [5, 13]. Fortunately, all the PMIS children in our study made a full neurologic recovery without sequelae. The absence of sNfL elevation in children confirms that neurologic involvement in post-COVID-19 PMIS is rather benign and the outcome is better than in neurologically impacted adults with COVID-19.

The study has several limitations. First, access to SARS-CoV-2 testing was variable by region and limited particularly during the early phase of the pandemic. Second, the number of PMIS patients in our study is limited (*n* = 14) and PMIS severity low; therefore, additional studies are warranted to investigate neuroaxonal integrity in those children. Third, in all participants, blood collection was done only at one timepoint; thus, the here reported results do not reflect sNfL dynamics which have higher predictive power than single point of time determinations, e.g., in Alzheimer’s disease or children with poor neurodevelopmental outcome [14, 15].

In conclusion, based on the deployment of sNfL as a biomarker for neuronal damage in a large pediatric population with asymptomatic to moderate COVID-19, there is no evidence that SARS-CoV-2 impacts neuroaxonal integrity in infected children.

## Data Availability

On request.
